# Annotations

**Published:** 1911-09-30

**Authors:** 


### ANNOTATIONS.

### A Case of Hydrophobia.

The abuse with which Mr. Walter Long was loaded a few years ago, at the date of the famous "muzzling order," has long ago subsided, to be replaced by the gratitude of the whole community, and of dog-lovers more especially. For it is universally admitted that that order, together with the quarantine regulations for dogs brought into the United Kingdom from abroad, stamped out rabies and hydrophobia most effectively; so that these two horrible diseases have been unknown among us for some years. It is, therefore, with distinct misgiving that the thoughtful section of the public will have received the information recently published as to the death of the Master of the South and West Wilts Foxhounds from hydrophobia, contracted, it is believed, from the bite of a fox. How a fox of home birth and breeding could contract rabies is a mystery; but if the facts are as stated, it is clear that this must somehow have happened. If one fox had the disease, it will be astonishing if no other cases of infection arise among the vulpine population; and if they do arise, it is quite certain that sooner or later there will be an outbreak among foxhounds or terriers. The one consoling feature about the sad tragedy of the M.F.H.'s death is that the bite supposed to be responsible for the infection was incurred a year ago.

### The Coroner's Heart.

Several papers have given prominence to the remarks of a Coroner upon his rejection for life insurance on account of a "weak heart." Twenty-three years later the Coroner went to another physician, who told him that his heart was perfectly healthy and accepted him for life insurance. It is often difficult to understand what doctors mean when they tell a patient that weakness of the heart is present. This diagnosis does not appear to be confined to the hearts of adults. A parent is not very uncommonly informed that the heart of a child is weak. The diagnosis of "weak heart" is responsible for much inconvenience and, it may be, misery. The opinion given that the heart is weak never leaves the minds of some people, and makes them, during possibly a long life, in some degree a burden to themselves and to their friends. Such an opinion too may alter a man's whole career. It is to be feared that the diagnosis of weakness of the heart is often—at least where examination for life insurance is concerned—the outcome of uncertainty. The medical man hears some sound which he considers abnormal. He does not feel prepared to diagnose definite cardiac disease, and is afraid lest subsequent events should prove his opinion, that the heart is healthy, to have been wrong. He therefore steers a middle course and describes the heart as "weak." Apart from life insurance, the diagnosis "weak heart" is no doubt frequently made where there is functional disturbance, and in children it seems probable that the presence of a diastolic sound at the apex may mislead. The common presence of such a sound does not seem to be generally recognised. A writer who should be considered an authority has recently stated that the diastolic sound invariably indicates the presence of early mitral stenosis. Such a statement is very far from the truth, and those who hold such an opinion must frequently give entirely unnecessary alarm to parents.

### Sea-Water Injections.

Three weeks ago attention was drawn in these columns (The Hospital, September 9, p. 587) to the fantastic and illogical principles on which Quinton's sea-water injection treatment is based, and to the need for caution in accepting for gospel the exuberant outpourings of lay journalists concerning the establishment at which this treatment is being administered free in London. An excellent description of the working of the "Quinton Polyclinic" as it appeared to a professional visitor, is contained in the current number of the *Guy's Hospital Gazette*, dated September 16. This record of personal inspection is obviously written by one who preserves the proper spirit of scientific scepticism; yet there is nothing in it which betrays bias or prejudice. The account given, and the comments expressed, should be read by anyone who contemplates sending or recommending any patient for this treatment, for they more than bear out those philosophic doubts which were here expressed as to the *rationale* of the sea-water treatment.

### Dichotomy on the Stage.

The Grand-Guignol, a theatre which caters essentially for those with a taste for the morbid and unpleasant, has recently presented a piece entitled "Dichotomie," in which the French medical profession appears in none too favourable a light. A certain Dr. Durtal, possessed of an ambitious wife anxious at all costs to become rich, has a wealthy patient who is suffering from a breast tumour. The illustrious surgeon Précigné will receive thirty thousand francs if he operates, of which sum one half will go to Durtal. Histological examination has failed to make out the cancerous nature of the tumour, and though Durtal is convinced that no operation is necessary he is persuaded by his wife to send his patient to the surgeon. The operation is decided upon, and preparations for the operation, instruments, and the patient are all seen on the stage. The spectators listen to the cries of the patient being subjected to chloroform, and the surgeon enters with blood on his arms. The patient has died during the performance of the operation. The Syndicat Médical de Paris has addressed to the *Matin*, through its secretary, Dr. G. Berruyer, a resolution in which it states that dichotomy is very exceptional, and that the body medical has always severely disapproved of it. Nevertheless it exists, and the morbidness of the play at the Grand-Guignol will convince the lay public that it is an important factor in the rise of the consultant.

